# Antler stem cells effectively alleviate the symptoms of cerebral ischemic injury via immunomodulation of the spleen

**DOI:** 10.1038/s41420-026-03218-4

**Published:** 2026-06-24

**Authors:** Si Wu, Yu Gao, Shunshun Zhong, Qianqian Guo, Yan Zhang, Huiying Li, Jiping Li, Huiling Luo, Hongyu Zhang, Qing Leng, Yufang Shi, Zhonghui Liu, Chunyi Li, Guangxian Nan

**Affiliations:** 1 Department of Neurology, China-Japan Union Hospital of Jilin University, Changchun, P. R. China; 2 Institute of Antler Science and Product Technology, Changchun Sci-Tech University, Changchun, P. R. China; 3 Department of Immunology, College of Basic Medical Sciences, Jilin University, Changchun, P. R. China; 4 Department of Cardiology, China-Japan Union Hospital of Jilin University, Jilin Provincial Cardiovascular Research Institute, Changchun, P. R. China; 5 The Fourth Affiliated Hospital of Soochow University, Institutes for Translational Medicine of Soochow University, Suzhou, P. R. China

**Keywords:** Stem cells, Immunology

## Abstract

Ischemic stroke is a major cause of disability and mortality worldwide. Increasing evidence indicates that peripheral immune responses contribute to secondary brain injury after stroke. The impact of the splenic response on brain injury has been well documented in animal models. This phenomenon remains poorly defined in the clinical situation. Through a retrospective analysis of 76 patients with acute anterior circulation large-vessel occlusion, we provide novel evidence that post-stroke spleen volume reduction is an outstanding risk factor for cerebral edema expansion, an association partially explained by a concomitant decrease in peripheral lymphocytes. Previous studies have shown that bone marrow-derived mesenchymal stem cells (BMSCs) improve stroke outcomes through immunomodulation peripherally. Nonetheless, efforts to identify more potent MSCs have never ceased. Antler stem cells, known for their potent regenerative and anti-inflammatory properties, represent promising candidates for stroke treatment. Using the transient middle cerebral artery occlusion (tMCAO) model rats, we found that intravenous administration of antler reserve mesenchymal cells (aRMCs, one type of antler stem cells) significantly improved motor function and reduced both cerebral edema and infarct volume, effects that were superior to those observed with BMSC treatment. Notably, these beneficial effects of aRMCs were attenuated in the splenectomized animals. Compared with the model and BMSC groups, the spleens of aRMC-treated rats exhibited increased weight, restored T cell populations, reduced lymphocyte activity, and downregulated expression of T cell migration-related genes. Additionally, these rats displayed decreased T cell infiltration into the cerebral infarct region. Mechanistically, the inhibitory effect of aRMCs on tMCAO-induced splenic T-cell migration is highly likely to be achieved via downregulation of CCR5 expression. These findings demonstrate a causal link between the degree of splenic contraction and the severity of brain edema, suggest spleen-to-brain T-cell migration as a therapeutic target, and position aRMCs as a promising and novel stem cell type for post-stroke neuroprotection with superior efficacy over BMSCs.

**Figure:**
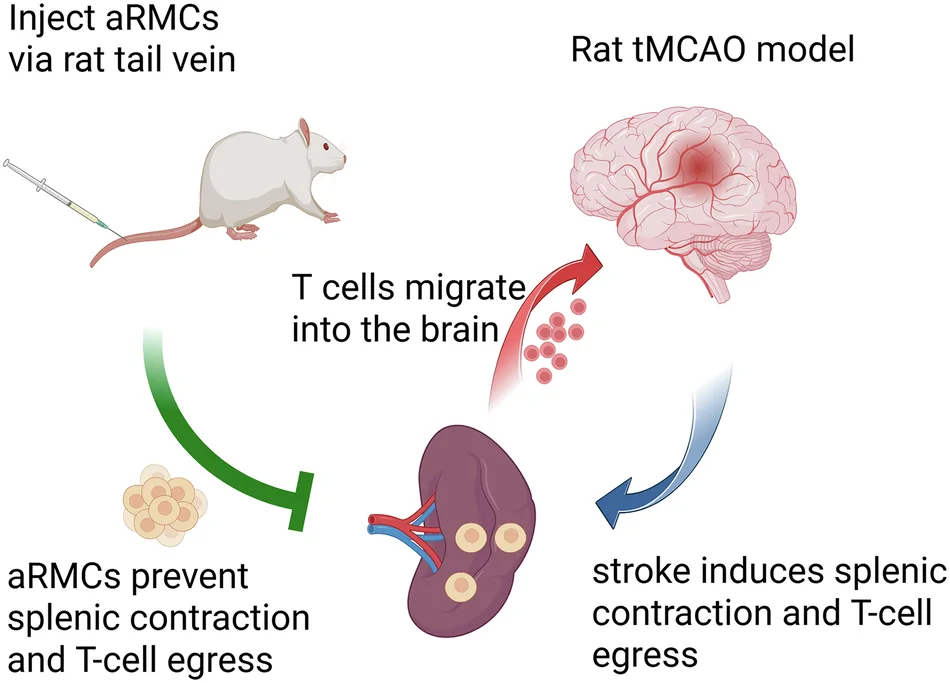

## Background

Ischemic stroke remains a major contributor to global mortality and morbidity [1]. Although reperfusion therapies have improved outcomes in many cases [2], substantial brain damage remains irreversible in most stroke survivors, highlighting the urgent need for novel treatment strategies. Accumulating evidence indicates that the peripheral immune system, particularly the spleen, is rapidly mobilized following ischemic stroke and contributes to post-ischemic cerebral damage in animal models [3–5]. Specifically, spleen-derived immune cells mobilized following splenic contraction have been shown to exacerbate cerebral ischemic injury [4, 5]. However, whether these splenic changes lead to clinically relevant events, such as cerebral edema expansion, remains poorly defined.

Stem cell-based therapies represent a promising approach for stroke treatment, with preclinical studies demonstrating their efficacy through mechanisms including cell replacement, trophic support mediated by secreted exosomes, and immunomodulation [6–8]. Given the limited ability of intravenously administered stem cells to enter the brain, immunomodulation rather than direct neuronal cell replacement has been recognized as a key mechanism underlying the effects of stem cell-based therapy [9, 10]. Bone marrow-derived mesenchymal stem cells (BMSCs) are the most extensively used MSC type in preclinical stroke models and have demonstrated immunomodulatory efficacy [11–13]. Nonetheless, seeking a more potent type of stem cell for the treatment of post-stroke cerebral damage would be one of the effective ways for a better clinical outcome.

Antler reserve mesenchymal cells (aRMCs), derived from the growth center of deer antlers, are a type of antler stem cells, characterized by their greatest regeneration potential compared with other types of stem cells [14, 15]. Moreover, the conditioned medium from aRMCs has been shown to exert potent anti-inflammatory and immunomodulatory effects [16]. These properties make aRMCs particularly promising for modulating peripheral splenic immune responses following ischemic stroke, hence better treating ischemic damage after stroke. The aim of the present study was twofold: (1) to evaluate the causal relationship between the post-stroke splenic contraction and ischemic stroke damage, and (2) to investigate the therapeutic potential of aRMCs compared with the BMSCs using a rat model of ischemic stroke.

## Results

### Reduced spleen volume was an outstanding risk factor for cerebral edema expansion in stroke patients

Among the 76 patients with acute anterior circulation large-vessel occlusion stroke included in this study, 34 (44.7%) exhibited cerebral edema expansion. Univariate analysis showed that, compared with patients without edema expansion, patients in the edema expansion group had a lower proportion of previous cerebral infarction/transient ischemic attack (TIA) (14.71% vs. 35.71%, *p* = 0.039), significantly lower peripheral blood lymphocyte count (0.96 × 10⁹/l [IQR 0.69, 1.63] vs. 1.45 × 10⁹/l [IQR 1.01, 2.01], *p* = 0.008), a higher admission NIHSS score (17 [IQR 14.75, 20] vs. 14 [IQR 12, 17.25], *p* = 0.007), and a significantly higher proportion of spleen volume reduction (52.94% vs. 16.67%, *p* = 0.001) (Table 1).

**Table 1:** Main parameters of patients with and without edema expansion.

	Edema expansion (*n* = 34)	No edema expansion (*n* = 42)	Z/t, X²	*p*-value
**Baseline characteristics**				
Age, yrs	63.71 ± 10.46	68.45 ± 11.43	1.869	0.066
Male sex, *n* (%)	22 (64.71%)	30 (71.43%)	0.393	0.531
**Clinical characteristics**				
Hypertension, *n* (%)	16 (47.06%)	23 (54.76%)	0.446	0.504
Diabetes, *n* (%)	11 (32.35%)	10 (23.81%)	0.686	0.408
Atrial fibrillation, *n* (%)	7 (20.59%)	9 (21.43%)	0.008	0.929
Cerebral infarction/TIA, *n* (%)	5 (14.71%)	15 (35.71%)	4.277	0.039
Blood total WBC count (×10⁹/l)	8.10 (IQR 6.84, 11.22)	9.09 (IQR 6.9, 11.27)	−0.517	0.605
Blood neutrophil count (×10⁹/l)	6.86 (IQR 5.33, 8.98)	6.85 (IQR 4.78, 8.27)	−0.616	0.538
Blood lymphocyte count (×10⁹/l)	0.96 (IQR 0.69, 1.63)	1.45 (IQR 1.01, 2.01)	−2.638	0.008
Blood monocytes count (×10⁹/l)	0.36 (IQR 0.22, 0.49)	0.39 (IQR 0.29, 0.53)	−1.288	0.198
NIHSS-admission	17 (IQR 14.75, 20)	14 (IQR 12, 17.25)	−2.69	0.007
Spleen volume reduction, *n* (%)	18 (52.94%)	7 (16.67%)	11.2	0.001

To adjust for confounding factors, variables with *p* < 0.10 in the univariate analysis, including age, history of cerebral infarction/TIA, lymphocyte count, NIHSS score, and spleen volume reduction, were entered into a binary logistic regression model. The results showed that spleen volume reduction was still a non-negligible risk factor for cerebral edema expansion (odds ratio [OR] = 4.980, 95% CI: 1.520–16.321, *p* = 0.008). Admission NIHSS score was also independently associated with cerebral edema expansion (OR = 1.130, 95% CI: 1.012–1.261, *p* = 0.029). Although peripheral blood lymphocyte counts did not reach statistical significance (OR = 0.473, 95% CI: 0.219–1.022, *p* = 0.057), the trend suggested a potential involvement of reduced lymphocyte count in cerebral edema expansion (Table 2).

**Table 2:** Binary logistic regression analysis of brain edema expansion.

Covariate	OR value	95% CI	*p*-value
Age	0.959	0.911, 1.009	0.108
Cerebral infarction/TIA	0.478	0.130, 1.759	0.267
Blood lymphocyte count	0.473	0.219, 1.022	0.057
NIHSS-admission	1.130	1.012, 1.261	0.029
Spleen volume reduction	4.980	1.520, 16.321	0.008

To further explore the relationship between spleen volume reduction and lymphocyte counts, we analyzed the clinical parameters of patients with or without spleen volume reduction (Supplementary Table 1). Compared with patients without spleen volume reduction, those with reduction had a significantly lower peripheral blood lymphocyte count (0.93 × 10⁹/l [IQR 0.75, 1.53] vs. 1.35 × 10⁹/l [IQR 0.96, 1.96], *p* = 0.033) and a higher proportion of cerebral edema expansion (72% vs. 31.37%, *p* = 0.001).

To evaluate the role of lymphocyte reduction in the contribution of spleen volume reduction to cerebral edema expansion, a binary logistic regression analysis was performed again with lymphocyte count removed from the model (Supplementary Table 2). The OR for spleen volume reduction increased to 5.862 (95% CI: 1.872–18.358, *p* = 0.002), suggesting that lymphocyte reduction may partially explain the association between spleen volume reduction and degree of cerebral edema expansion.

Taken together, these findings indicate that acute spleen volume reduction after ischemic stroke is an outstanding risk factor for cerebral edema expansion and that this effect may be partially explained by a reduction in the number of peripheral lymphocytes.

### aRMC administration effectively improved outcomes of stroke-induced damage

**Fig. 1:**
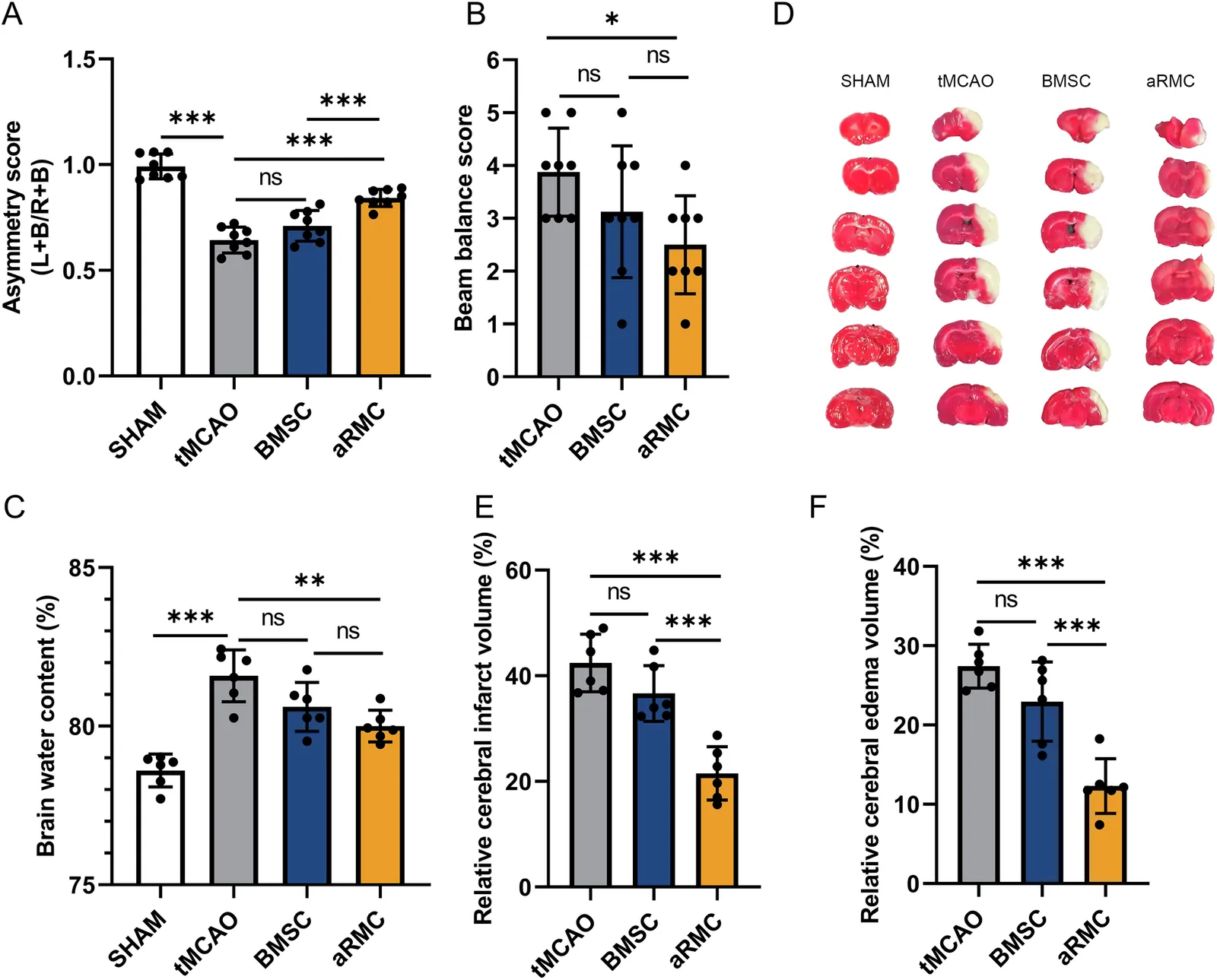
Assessment of behavioral outcomes, infarct volume, and brain water content. **A** Functional recovery assessed using the cylinder test (*n* = 8). B, both left and right forelimbs; L left forelimb, R right forelimb. **B** Functional recovery assessed using the beam balance test (*n* = 8). **C** Brain water content assessment (*n* = 6). **D** Representative TTC-stained brain sections. **E**, **F** Relative cerebral infarct volume and cerebral edema volume were calculated based on TTC staining (*n* = 6). Data are expressed as mean ± SD. *p < 0.05, **p < 0.01, ***p < 0.001, ns non-significant.

Motor function was assessed using the cylinder test and beam balance test. In the cylinder test, model rats showed a significant increase in forelimb asymmetry, indicating greater reliance on the unaffected limb. Notably, aRMC-treated rats showed significantly improved performance compared with those in the model and BMSC-treated groups, whereas no significant difference was observed between the model and BMSC-treated groups (Fig. 1A). Similarly, in the beam balance test, the aRMC-treated rats exhibited significantly better motor coordination and balance than those from the model group, whereas the BMSC-treated group showed a non-significant improvement compared with the model group (Fig. 1B).

Infarct volume was assessed by 2,3,5-triphenyltetrazolium chloride (TTC) staining, and brain water content was quantified using the wet-dry method. Compared with the model group, the BMSC group showed a reduction in infarct volume and brain edema that did not reach statistical significance. In contrast, the aRMC group significantly reduced both parameters compared with the model group. Moreover, the aRMC group was significantly superior to the BMSC group (Fig. 1C–F).

### aRMC administration effectively reversed the reduced spleen weight induced in the model group

**Fig. 2:**
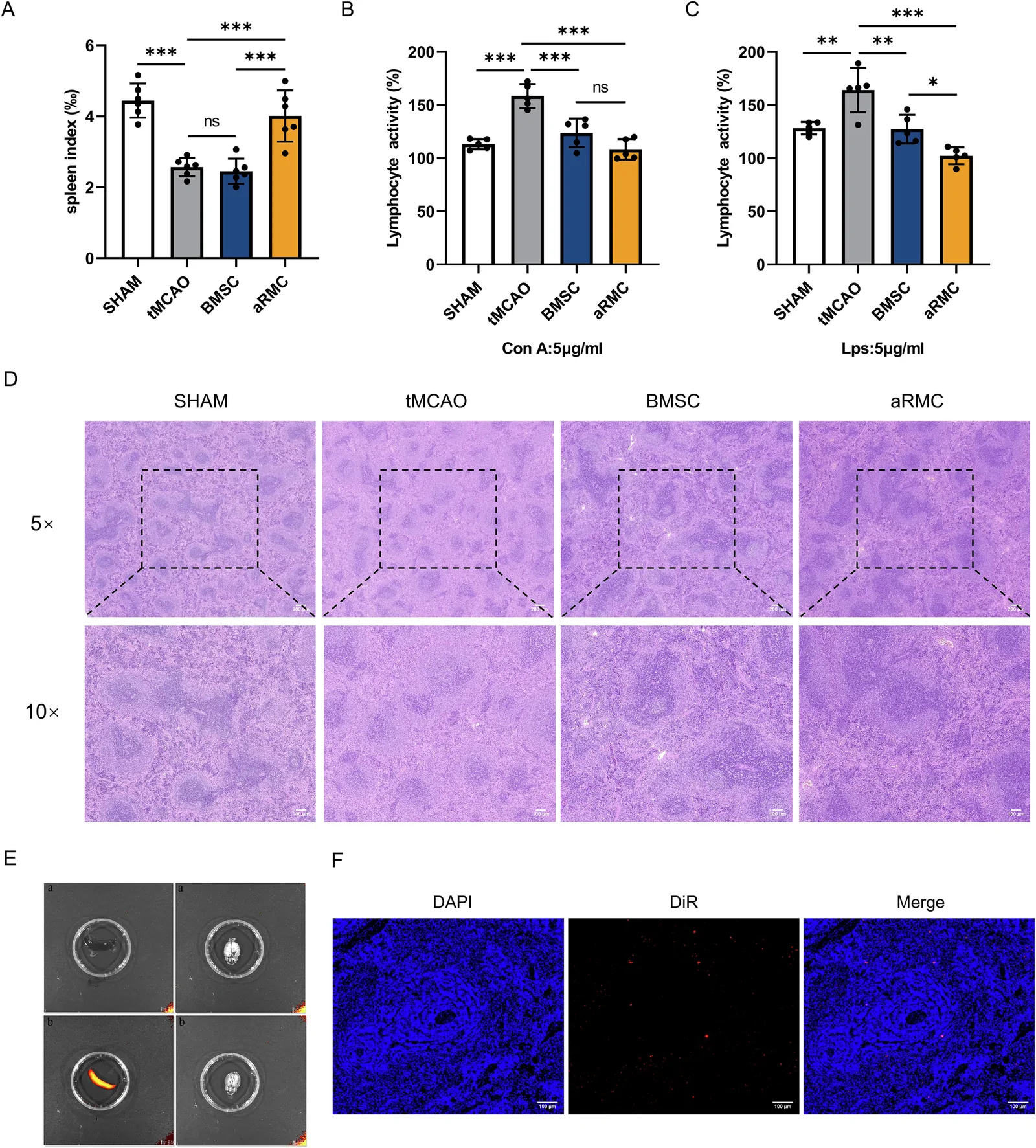
Splenic alterations and distribution of aRMCs after ischemic stroke. **A** Spleen index (spleen weight/body weight ratio; *n* = 6). **B**, **C** Lymphocyte activity after stimulation with Con A (5.0 μg/ml) or LPS (5.0 μg/ml) (*n* = 5). **D** H&E staining of the spleen. Compared with the sham and aRMC groups, the model and BMSC groups exhibited follicular atrophy and lymphocyte depletion in the periarteriolar lymphoid sheath (*n* = 4). Scale bar = 200 µm for 5× and 100 µm for 10× magnification. **E** Distribution of aRMCs in the organs of both brain and spleen (a. non-labeled aRMCs; b. DiR-labeled aRMCs). **F** Fluorescence images showing engrafted aRMCs in the spleen (*n* = 3). Scale bar = 100 µm. Data expressed as mean ± SD. *p < 0.05, **p < 0.01, ***p < 0.001, ns non-significant.

Spleens were harvested from all groups to verify their changes. The spleen index of rats in both the model and BMSC groups was significantly lower than that of the sham-operated group; however, that of the aRMC group was not significantly different from the sham group (Fig. 2A), indicating that aRMCs, unlike BMSCs, effectively restored post-stroke splenic atrophy.

### aRMC administration effectively suppressed the tMCAO-induced lymphocyte activation

Splenic lymphocyte activation was assessed by the CCK-8 assay. The results showed that Con A (5.0 µg/ml) significantly increased T lymphocyte activation in the model group compared with that in the sham group; this increase was significantly inhibited by the BMSC treatment (*p* = 0.0004) and more greatly by the aRMC treatment (*p* < 0.0001) (Fig. 2B). Similarly, B lymphocyte activation stimulated by LPS (5 µg/ml) was elevated: compared with the tMCAO group, this activation was significantly attenuated in both the BMSC and aRMC groups (Fig. 2C).

### aRMC administration effectively attenuated tMCAO-induced histopathological alterations in the spleen

Histological examination of the collected spleen tissue revealed follicular atrophy and lymphocyte depletion in the periarteriolar lymphoid sheath in the model group. These pathological changes were significantly alleviated in both the BMSC and aRMC groups, with a more pronounced improvement observed in the aRMC group (Fig. 2D).

### DiR-labeled aRMCs were predominantly distributed in the spleen rather than in the brain

Next, we performed a cell-tracing experiment using DiR-labeled aRMCs. In vivo and ex vivo fluorescence imaging analyses were conducted using the IVIS Spectrum system to determine the distribution of aRMCs. No positive signal was detected in the in vivo imaging analysis. Ex vivo imaging analysis confirmed their specific distribution in the spleen, but with no detectable signal in the brain (Fig. 2E). Immunofluorescence analysis of spleen sections further corroborated this finding, demonstrating that the DiR-positive aRMCs were predominantly lodged within the periarterial lymphatic sheath (Fig. 2F).

### Spleen was essential for the protective effects of aRMCs against stroke damage

To evaluate the role of the spleen in the observed functional improvement in the aRMC-treated group, a new animal model was established in which the spleen was excised two days prior to the treatment of tMCAO. The results showed that there was no significant difference in behavior or histopathology detected between the aRMC group and the model group in the spleen-less rats (Supplementary Fig. 1). These results suggest that the intravenously administered aRMCs can effectively improve neurological function only in the presence of a spleen.

### mRNA sequencing of splenocytes further supported our foregoing conclusion

**Fig. 3:**
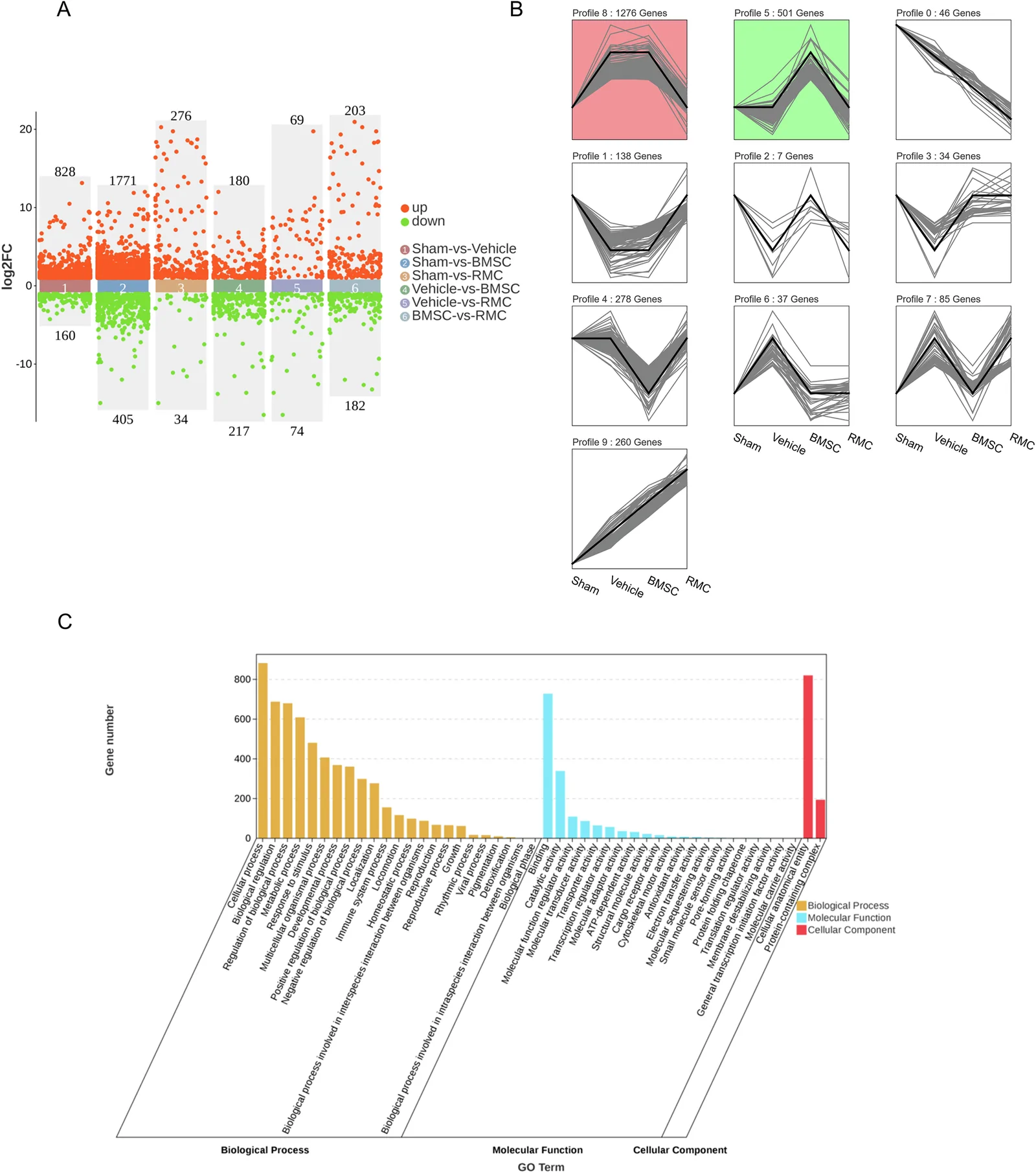
mRNA sequencing analysis of the spleen (*n* = 3). **A** Scatter plots of DEGs in the spleen among the sham, model, BMSC, and aRMC groups. In pairwise comparisons, red and green dots represent significantly upregulated (log2FC > 1) and downregulated (log2FC < −1) genes, respectively. **B** STEM analysis identified 10 expression profile modules, among which profile 8 (containing 1276 genes) showed a significant expression pattern characterized by high expression in the model and BMSC groups and low expression in the sham and aRMC groups, consistent with the phenotypic changes observed in the spleen index. **C** GO analysis of profile8 genes categorized by biological process, molecular function, and cellular component.

To further investigate along this line, we conducted mRNA sequencing of the spleen to evaluate the changes in gene expression profiles. The scatter plot showed that among the different groups, profiles of the differentially expressed genes (DEGs) of the aRMC group clustered more closely with the sham group than with the BMSC group (Fig. 3A). Using STEM analysis, we identified 10 significant expression profile modules, among which Profile8 and Profile5 modules were significantly enriched. Subsequently, the Profile8 module, which showed a consistent trend with phenotype, was selected for further analysis (Fig. 3B). To investigate the biological functions of the genes in the Profile8 module, Gene Ontology (GO) enrichment analysis was performed. As shown in Fig. 3C, the enriched biological processes include cellular processes, biological regulation, metabolic processes, and responses to stimuli. In terms of molecular function, binding is the most enriched, followed by catalytic activity and molecular function regulator activity. Regarding cellular components, enrichment is observed in cellular anatomical entities and protein-containing complexes.

**Fig. 4:**
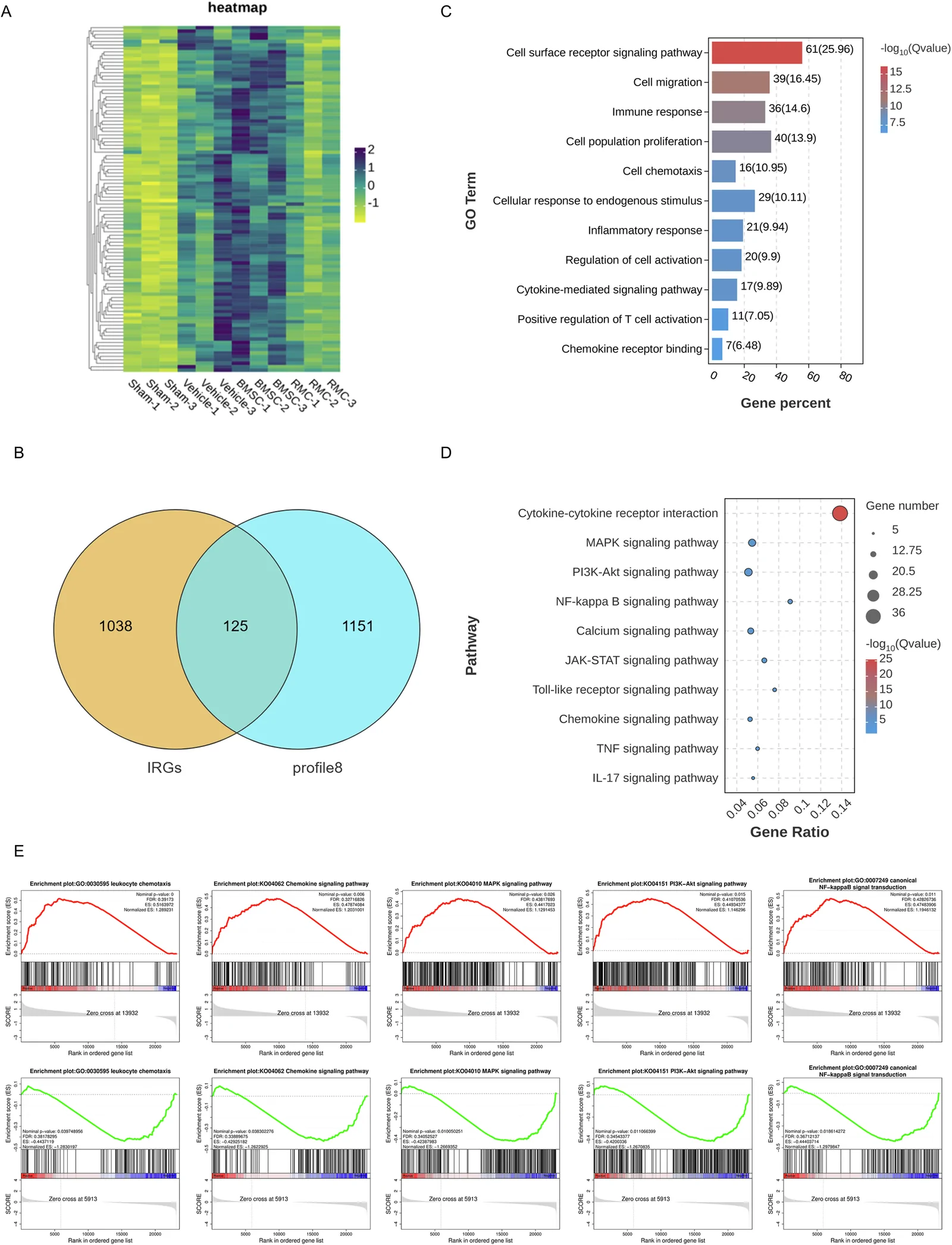
Bioinformatic analysis of IRGs in profile 8. **A** Heatmaps showing distinct mRNA expression profiles among samples from the different groups. **B** Venn diagram illustrating the overlap between profile8 genes and IRGs from the IMMPORT database. **C**, **D** GO and KEGG analysis of IRGs in profile8. **E** GSEA performed on all genes in the comparative groups. Each pair of plots shows the enrichment score across the ranked gene list, with peaks indicating significant enrichment of specific pathways. (The red line represents model vs. sham, and the green line represents aRMC vs. model.).

Heatmaps exhibited distinct differential expression profiles among the four group samples (Fig. 4A). From these results, we aimed to identify key immune-related genes, which were obtained from the IMMPORT database. A Venn diagram showed the intersection of Profile8 DEGs and immune-related genes (IRGs), revealing 125 immune-related DEGs (Fig. 4B). Gene Ontology Biological Process (GOBP) analysis of these 125 immune-related DEGs showed that they were primarily involved in processes such as cell surface receptor signaling, cell migration, immune response, cell population proliferation, and cell chemotaxis (Fig. 4C). Subsequent KEGG enrichment analysis revealed that these genes were significantly enriched in signaling pathways related to cell proliferation and inflammation-related signaling pathways, including cytokine-cytokine receptor interaction and the MAPK, PI3K-AKT, and NF-κB signaling pathways (Fig. 4D). To gain further insight into the underlying pathways, we conducted GSEA, which demonstrated significant enrichment of gene sets critically involved in leukocyte chemotaxis, as well as the chemokine, MAPK, PI3K-AKT, and NF-κB signaling pathways (Fig. 4E). These findings underscore that the splenic response to ischemia is linked to inflammation responses and cell chemotaxis, and that aRMC treatment effectively attenuates these processes via modulating the relevant signaling pathways.

### aRMC administration rescued the reduction of splenic T cells induced by tMCAO

**Fig. 5:**
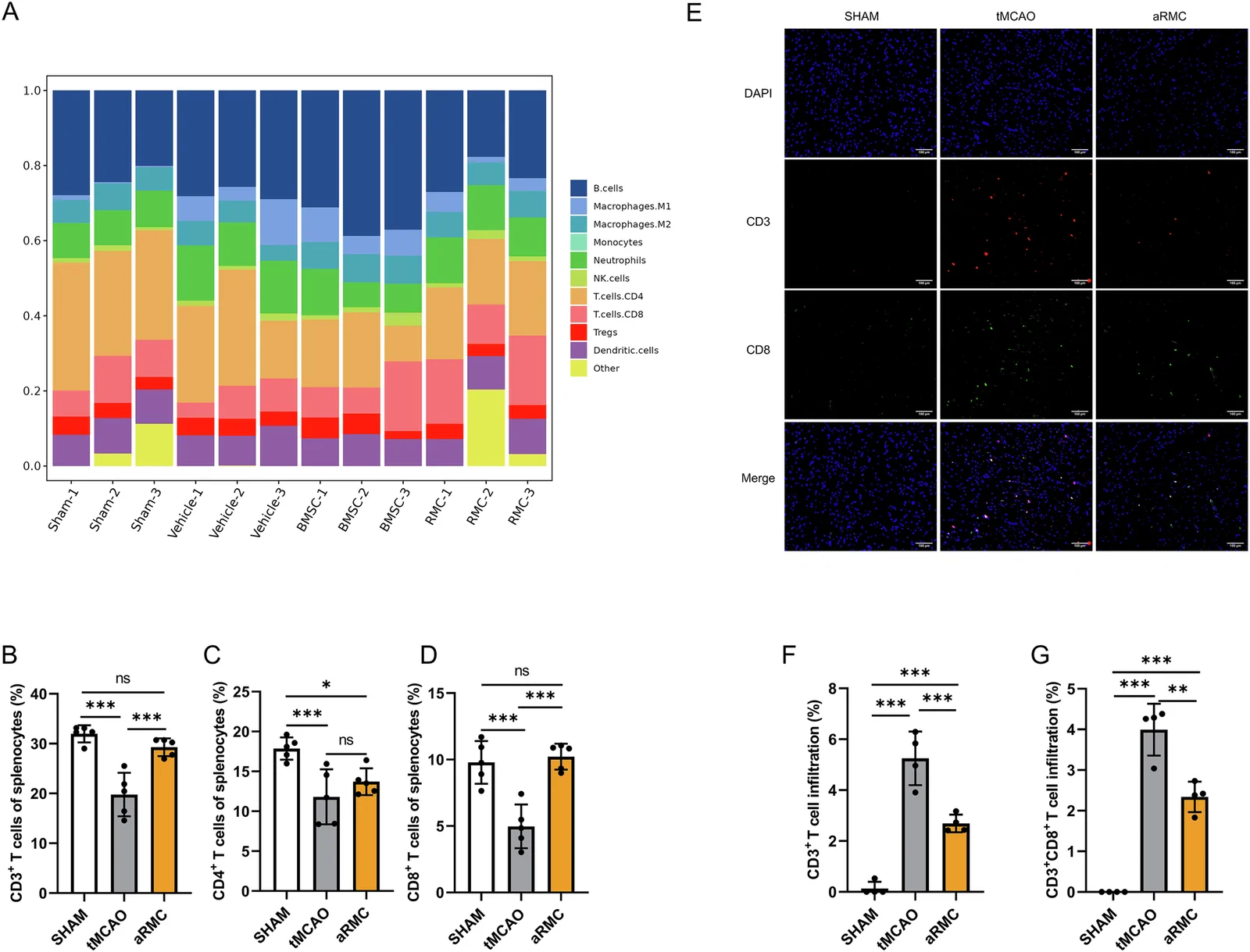
Splenic immune cell composition and brain T-cell infiltration. **A** Stacked bar chart generated by quanTIseq based on mRNA-sequencing data (*n* = 3). **B** Flow cytometric analysis of CD3^+^ T cells among splenocytes (*n* = 5). **C** Flow cytometric analysis of CD3^+^CD4^+^ T cells among splenocytes (*n* = 5). **D** Flow cytometric analysis of CD3^+^CD8^+^ T cells among splenocytes (*n* = 5). **E** Immunofluorescence analysis of CD3^+^CD8^+^ T-cell infiltration in cerebral infarct region (*n* = 4; scale bar = 100 μm). **F** Quantification of CD3^+^ T-cell infiltration (*n* = 4). **G** Quantification of CD3^+^CD8^+^ T-cell infiltration (*n* = 4). Data expressed as mean ± SD. *p < 0.05, **p < 0.01, ***p < 0.001, ns non-significant.

Due to the fact that the reduction in spleen weight after stroke is primarily attributable to the mobilization of immune cells into the peripheral circulation, we estimated the relative abundance of the ten immune cell types from mRNA-sequencing data using the quanTIseq method. This analysis revealed that stroke predominantly induced a reduction in splenic T-cell numbers, which was then rescued by the aRMC treatment, whereas BMSC treatment had no significant effect detected (Fig. 5A).

Flow cytometry was subsequently performed to analyze the proportions of T cells, neutrophils, and monocytes in the spleen. The results showed that tMCAO treatment primarily induced a reduction in T cells, specifically in the CD8^+^ T-cell subset, and that aRMC treatment effectively rescued this reduction (Fig. 5B–D). No significant differences in the proportions of neutrophils and monocytes were observed among these groups (Suppl. Fig. 2A–C). These findings were consistent with those of the immune cell abundance results obtained from the mRNA sequencing.

### aRMC administration reduced T-cell infiltration into the brain

Immunofluorescent staining was performed to assess the degree of T-cell infiltration in the area of brain damage. Results demonstrated a significant increase in T-cell infiltration in the brain after tMCAO, which was significantly reduced by aRMC treatment (Fig. 5E, F). Notably, this reduction was mainly attributable to a decrease in the CD3^+^CD8^+^ T-cell subset (Fig. 5G), whereas no significant change was observed in the CD3^+^CD4^+^ T-cell subset (Supplementary Fig. 2D).

### aRMC administration inhibited tMCAO-induced splenic T-cell migration via downregulating CCR5

**Fig. 6:**
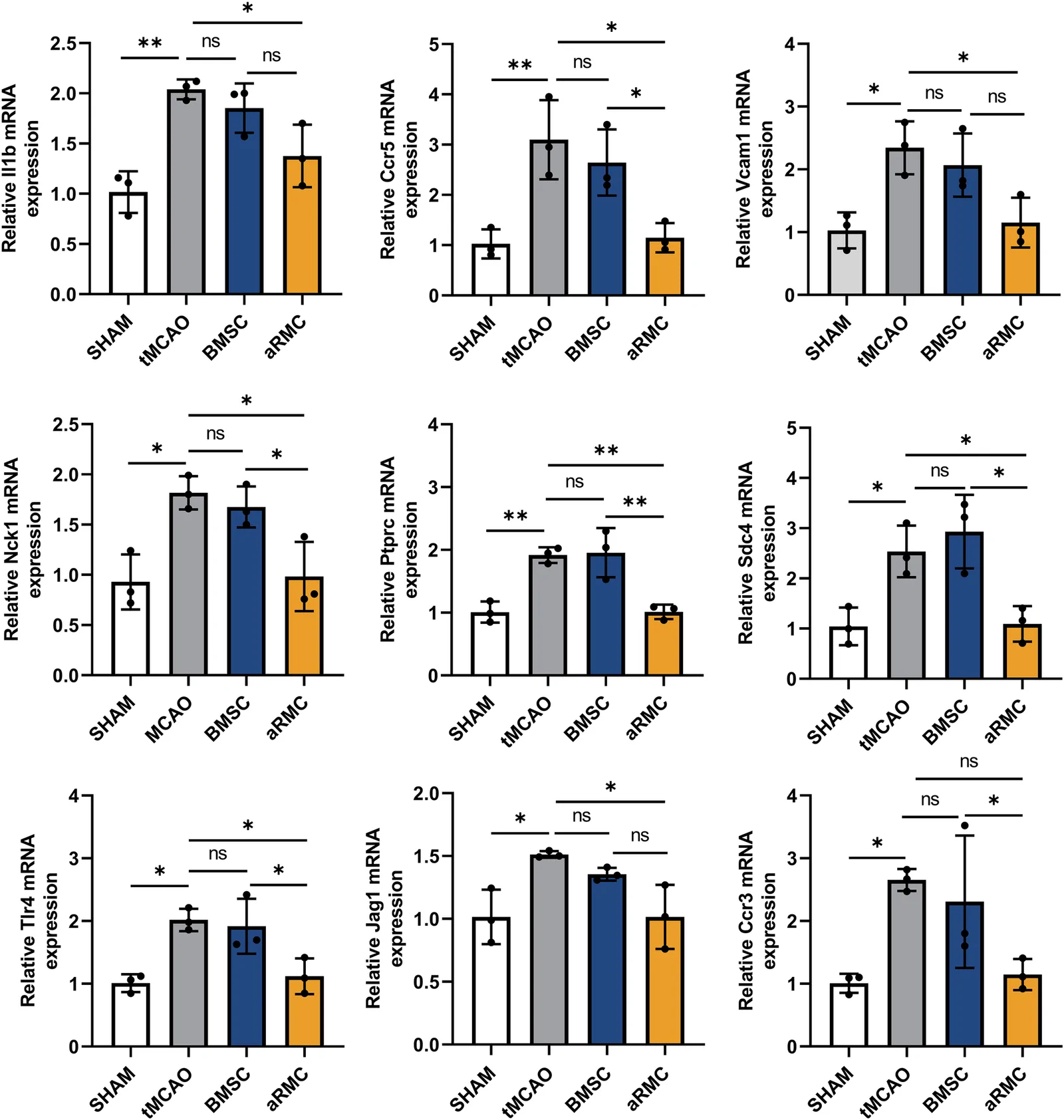
Expression levels of T-cell migration-related genes in the spleen (*n* = 3). Data expressed as mean ± SD. *p < 0.05, **p < 0.01, ***p < 0.001, ns non-significant.

Given that aRMC treatment restored splenic T-cell abundance and concomitantly reduced cerebral T-cell infiltration, we interrogated the mRNA-sequencing dataset to identify candidate genes implicated in T-cell migration. Validation by RT-qPCR revealed that the expression levels of T cell migration-related genes, including Ccr5, Ccr3, Il1b, Tlr4, Vcam1, Ptprc, Nck1, Jag1, and Sdc4, were significantly higher in the tMCAO group than in the aRMC group, whereas no significant difference was observed between the tMCAO and BMSC groups (Fig. 6; the primer sequences for these genes are provided in Suppl. Table 3).

**Fig. 7:**
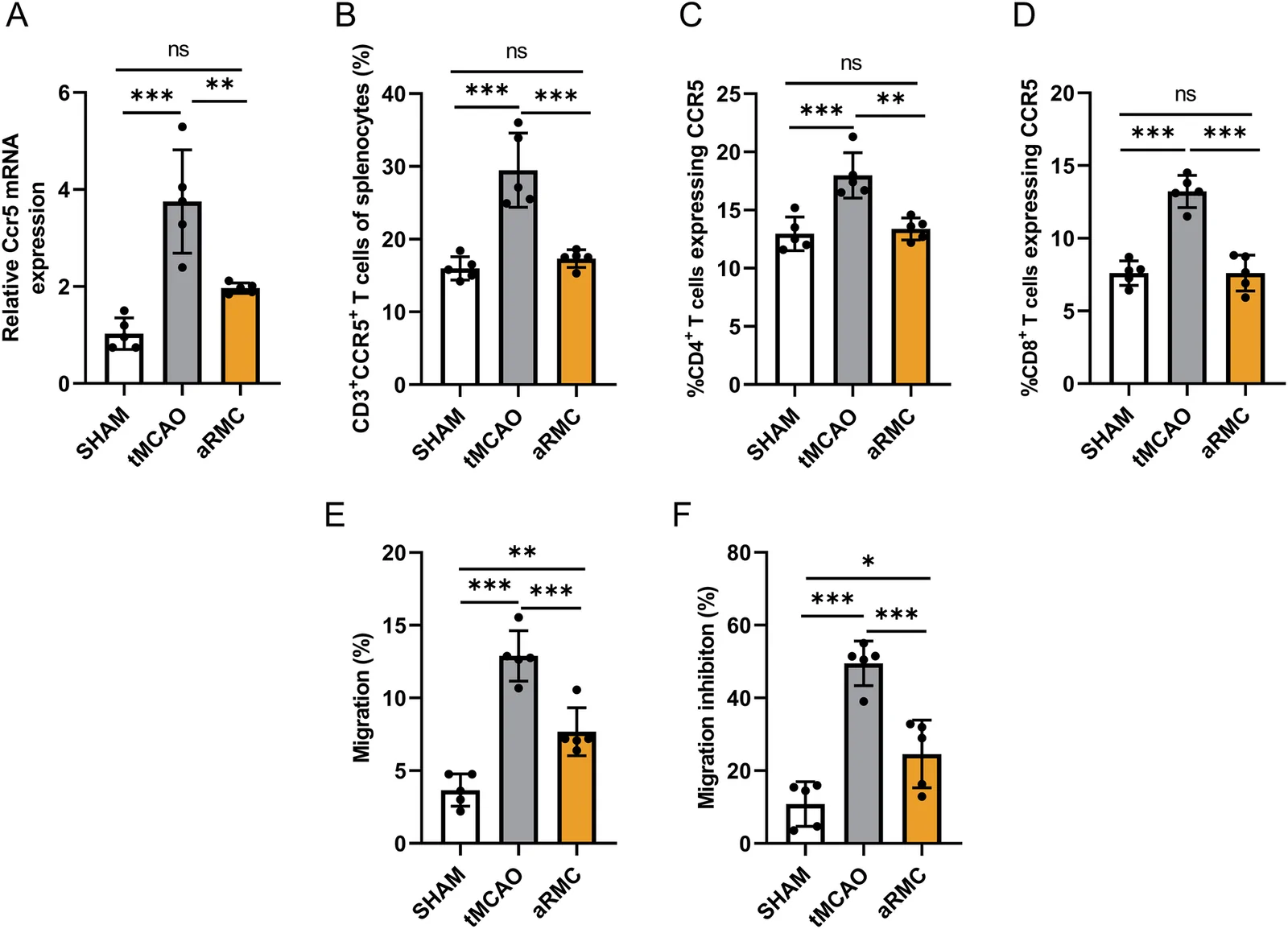
aRMCs suppress CCR5 expression in splenic T cells and reduce CCR5-dependent T-cell migration. **A** mRNA expression level of CCR5 in purified T cells (*n* = 5). **B** Percentage of CD3^+^CCR5^+^ T cells analyzed by flow cytometry (*n* = 5). **C**, **D** Proportions of CD4^+^CCR5^+^ T cells and CD8^+^CCR5^+^ T cells analyzed by flow cytometry (*n* = 5). **E** Migration of CD3^+^ T cells (*n* = 5). **F** Effect of maraviroc on CD3^+^ T-cell migration (*n* = 5). Data expressed as mean ± SD. *p < 0.05, **p < 0.01, ***p < 0.001, ns non-significant.

To investigate the underlying mechanism whereby aRMC treatment effectively inhibited splenic T-cell migration, T cells were isolated from the spleens of the sham, model, and aRMC groups, and with the purity of T lymphocytes being 92.4% (Supplementary Fig. 3). Both the RT-qPCR and flow cytometric analyses showed that CCR5 expression was upregulated in the splenic T cells after the treatment of tMCAO, and was subsequently attenuated following the aRMC treatment (Fig. 7A, B). Notably, aRMC treatment reduced the proportion of CCR5^+^ T cells in both the CD4^+^ and CD8^+^ T-cell subpopulations (Fig. 7C, D).

Next, we performed a Transwell migration assay to quantify T-cell migratory capacity. Compared with the sham group, the model group markedly increased the number of migrated T cells, whereas aRMC treatment significantly reduced T-cell migration compared with the model group (Fig. 7E). Blockade of CCR5 with the pharmacological chemical, maraviroc, robustly suppressed T-cell migration in the model group; in contrast, the magnitude of maraviroc-mediated inhibition was comparatively abolished in the aRMC-treated group (Fig. 7F).

## Discussion

Ischemic stroke triggers a rapid and systemic immune response that extends far beyond the injured brain [17]. Splenic contraction observed after stroke likely reflects this complex systemic response, involving neurohumoral activation and immune cell redistribution in animal studies [4, 18]. In this study, we provided evidence via analyzing the available data demonstrating that spleen volume reduction was an outstanding risk factor for cerebral edema expansion in patients with stroke. Building upon this analysis using clinical data, we further demonstrated in a rat tMCAO model that antler reserve mesenchymal cells (aRMCs), one type of antler stem cells, exerted superior neuroprotective effects over the bone marrow mesenchymal stem cells (BMSCs), a cell type classically used in the clinics. Mechanistically, aRMCs inhibited the migration of T cells, particularly CD8⁺ T cells, from the spleen to the ischemic brain via downregulation of CCR5 expression on splenic T cells.

Although splenic contraction has been observed in patients after stroke [19–23], whether this phenomenon is causally related to stroke and whether this contraction influences stroke severity remains unclear. To our knowledge, this is the first clinical study to identify acute-phase spleen volume reduction as an outstanding risk factor for cerebral edema expansion in stroke patients. Furthermore, we found that this association is partially explained by a concomitant reduction in peripheral lymphocytes. This relationship and underlying mechanism align with preclinical evidence demonstrating a critical link between the splenic response and the severity of brain injury [24–26]. Collectively, these findings highlight splenic modulation as a promising avenue for further investigation.

Previous studies have shown that antler stem cell-derived conditioned medium and exosomes exert anti-inflammatory and immunomodulatory effects in models of gingival inflammation [16], osteoarthritis [27], and pulmonary fibrosis [28]. BMSCs, as a well-studied and classical source of mesenchymal stem cells, have been reported to exert anti-inflammatory and neuroprotective effects in cerebral ischemic injury [11–13]. Here, we demonstrate for the first time that administration of aRMCs confers superior therapeutic efficacy over BMSCs in the animal stroke model. Notably, the effects of aRMC treatment were attenuated in the splenectomized rats, indicating that a functional spleen is required for the therapeutic effect of aRMCs. Consistently, evaluation of cell distribution showed that aRMCs preferentially lodged in the spleen rather than in the brain, suggesting that their neuroprotective effects are unlikely to be direct, but rather mediated by the peripheral immune organ, the spleen. These findings indicate that alleviation of post-stroke injury by aRMCs would be through modulating peripheral immune responses from the spleen. In line with this, aRMCs, but not BMSCs, rescued post-stroke splenic atrophy and restored T cell populations in the spleen. Transcriptomic profiling of splenic tissue further revealed that aRMCs effectively suppressed the immune activation program induced by tMCAO. Compared with BMSCs, aRMCs more effectively downregulated genes involved in cell migration, proliferation, and inflammatory responses, and more strongly attenuated key signaling pathways, including cytokine and chemokine cascades, as well as MAPK, PI3K-AKT, and NF-κB. These findings collectively demonstrate that aRMCs mitigate ischemic injury via indirect modulation of splenic immune responses.

Previous studies indicate that T cells play a deleterious role in the acute phase of ischemic stroke [4, 29], especially CD8^+^ T cells [30]. Although we did not directly track cell migration destination, the coordinated depletion of splenic T cells and their accumulation in infarct regions, together with previous studies that directly tracked fluorescently labeled splenocytes to the ischemic brain [31], strongly suggest that the spleen serves as a major source of T cells after stroke. We observed that tMCAO induced a marked reduction of splenic T cells and a concomitant increase in T-cell infiltration within the ischemic brain. Notably, aRMC treatment attenuated both the loss of splenic T cells and the infiltration of T cells (particularly CD8⁺ T cells) into the brain. These findings suggest that aRMCs ameliorate brain injury by attenuating the egress of T cells from the spleen and their subsequent infiltration into the brain.

Stroke creates a systemic chemotactic milieu for peripheral immune cells to migrate to the injured brain region [4, 32, 33]. Previous studies have established the role of CCR5 in mediating T-cell migration to sites of inflammation, including leishmanial lesions [34] and tumors [35]. Additionally, research has shown an increase in CCR5 expression in the spleen following the treatment of tMCAO [36]. In our study, CCR5 emerged as a prominent candidate among the differentially expressed immune-related genes, exhibiting increased expression in splenic T cells following tMCAO, particularly within the CD8⁺ subpopulation. aRMC treatment significantly attenuated CCR5 expression at both transcriptional and protein levels on T cells. Cell migration assays confirmed that T cell migration was, at least in part, CCR5-dependent. CCR5 blockade with the pharmacological agent maraviroc markedly reduced T cell migration. While other mediators may also contribute to T cell trafficking, our findings identify suppression of CCR5-associated migratory signaling as a key mechanism whereby aRMCs modulate splenic immune responses and limit cerebral immune cell infiltration.

Several animal studies have indicated that the spleen is a key effector organ in mediating the effects of intravenously delivered stem cells for the effective treatment of ischemic stroke. Mechanistically, diverse stem cell types, including multilineage-differentiating stress-enduring (Muse) cells [10], multipotent adult progenitor cells [37], umbilical cord blood cells [38], and hematopoietic stem cells [39], improve stroke outcomes through spleen-mediated immunomodulation, primarily by altering immune cell composition and downregulating proinflammatory cytokine and chemokine receptor genes in the spleen. Consistent with and extending these observations, our study demonstrates that intravenously administered aRMCs preferentially lodge in the spleen rather than the brain and inhibit tMCAO-induced splenic T-cell migration. Notably, aRMCs exhibited superior therapeutic efficacy over the well-established cell type, BMSCs, positioning this cell type not only as a new addition to this immunomodulatory paradigm but as a particularly promising candidate for the development of therapeutics.

Several limitations of this study should be acknowledged. First, a thorough quantitative assessment of the distribution of aRMCs across organs other than the brain and spleen was not performed. Second, although our mRNA sequencing data offer preliminary insights, it is important to note that modulating T-cell migration may be only one aspect of aRMC immunomodulatory functions. Further research is crucial to enhance our understanding of the roles of aRMCs and the underlying mechanisms in the context of treating cerebral infarction in the clinical setting.

## Conclusion

Our study demonstrates that stroke-induced splenic contraction contributes to secondary brain injury, potentially by promoting splenic T-cell migration into the brain. aRMC treatment attenuates ischemic brain injury highly likely via inhibiting splenic T-cell trafficking to the brain, an effect that may be partially mediated by CCR5. Together, these findings provide evidence that spleen-to-brain T-cell migration serves as an important therapeutic target for alleviating secondary injury after cerebral ischemia, and position aRMCs as a promising stem cell type for the therapy of ischemic stroke, with superior efficacy over BMSCs.

## Materials and methods

### Clinical data collection

A retrospective analysis included 76 patients with acute anterior circulation large-vessel occlusion who underwent successful emergency endovascular treatment. Clinical data were extracted from electronic medical records. All patients were admitted within 6 h of symptom onset. Splenic morphology was assessed using chest CT scans obtained at admission and 24 h later, and spleen volume was calculated using ImageJ software. Based on previous findings showing minimal day-to-day variation in spleen volume among healthy controls (with a maximum mean difference of 9.5 cm^3^ and an average change of 1.24 cm^3^) [19], a reduction in spleen volume ≥ 10 cm^3^ was defined as significant volume loss. The exclusion criteria included pre-existing hematological disorders; active infection within 1 week before stroke onset; use of immunosuppressive medications; active malignancy under treatment; autoimmune disease; and age < 18 years. Stroke severity was assessed using the National Institutes of Health Stroke Scale (NIHSS) at admission. Cerebral edema was evaluated using the Wardlaw score [40] based on head CT scans at admission and 24 h later. An increase in Wardlaw score ≥ 2 was defined as true cerebral edema expansion [41, 42]. The clinical study protocol was approved by the local Ethics Committee of China-Japan Union Hospital of Jilin University (Approval No. 2025121102). All methods and procedures performed were in accordance with relevant guidelines and regulations.

### Transient middle cerebral artery occlusion (tMCAO)

Male Sprague-Dawley (SD) rats (6–8 weeks old) were obtained from Yisi Laboratory Animal Technology Co., Ltd. (Changchun, China). All animals were housed under standard laboratory conditions with a 12 h light/dark cycle, a temperature of 22 ± 2 °C, and a relative humidity of 55 ± 5%, with ad libitum access to food and water. Transient focal cerebral ischemia was induced by 90 min of right middle cerebral artery occlusion using the intraluminal filament technique under isoflurane anesthesia, as previously described [43, 44]. Body temperature was maintained at 37 °C during surgery, and animals were allowed to recover at room temperature after the procedure. Sham-operated rats underwent the same surgical procedures except for filament insertion. All experimental procedures were approved by the Animal Ethics Committee of Jilin University (Approval No. 2025708) and performed in accordance with China’s national standard GB/T 35892–2018 “Guidelines for Ethical Review of Laboratory Animal Welfare”.

### Splenectomy

Splenectomy was performed as described in a previous study [45]. Rats were anesthetized with isoflurane, and a 1-cm incision was made in the left upper abdominal quadrant. The spleen was exteriorized, and the splenic hilum was ligated. The incision was then closed with a continuous suture. The animals were allowed to recover for 2 days before undergoing subsequent procedures [10].

### aRMC and bone marrow-derived mesenchymal stem cell (BMSC) treatments

To evaluate the therapeutic effects of aRMCs and BMSCs in a rat stroke model, rats were randomly assigned to four groups: sham-operated, model, BMSC-treated, and aRMC-treated groups. aRMCs were provided by the Institute of Antler Science and Product Technology (Changchun, China), and rat BMSCs were purchased from OriCell (Cat. No. RASMX-01001, Guangzhou, China). Immediately after reperfusion, rats in treatment groups received an intravenous injection of either BMSCs or aRMCs (1 × 10⁶ cells suspended in 1 ml saline) via the tail vein at a rate of 1 ml/min. Rats in the sham-operated and model groups received an equal volume of saline following the same injection protocol.

### Behavioral assessments

Neurological function was assessed 24 h after reperfusion using the cylinder test and the beam balance test by investigators blinded to group allocation. In the cylinder test, each rat was individually placed in a transparent glass cylinder (9 cm in diameter and 15 cm in height), and forelimb use during spontaneous rearing was recorded from the side. Forelimb use was scored until 20 movements had been recorded. The asymmetry score was calculated as (left forelimb contacts + contacts with both forelimbs)/(right forelimb contacts + contacts with both forelimbs) [10, 46]. Motor coordination and muscle strength were further assessed using the beam balance test as previously described [47]. Rats were pretrained to voluntarily traverse a narrow wooden beam (122 cm long, 2.5 cm wide, and 42 cm high) without slipping before testing. Performance was graded on a 0–6 scale according to established scoring criteria [47].

### Assessment of cerebral infarction and brain edema

Rats were deeply anesthetized with isoflurane and euthanized by decapitation 24 h after surgery. Brains were rapidly removed for infarct volume and brain edema assessments.

For infarct volume assessment, six consecutive 2-mm-thick coronal sections were prepared using a rodent brain matrix (RWD, Shenzhen, China). The sections were stained with 2% TTC stain solution (Coolaber, Beijing, China) at 37 °C for 15 min, followed by fixation in 4% paraformaldehyde (Servicebio, Wuhan, China) for 1 h at room temperature prior to photography. Images were analyzed using ImageJ software. For each coronal section, the areas of the contralateral hemisphere, the entire ipsilateral hemisphere, and the non-infarcted tissue within the ipsilateral hemisphere were measured. The infarct volume was then calculated as the sum of lesion areas across all six slices, according to the method described previously [48].

Brain water content was measured using the wet-dry method [49]. The brain was rapidly harvested, and the olfactory bulbs, cerebellum, and brainstem were removed. The cerebral hemispheres were then separated along the sagittal suture. The ipsilateral (right) hemisphere was immediately weighed using an electronic analytical balance to obtain the wet weight. The tissue was subsequently dried in an oven at 72 °C until a constant weight was reached (weight change < 0.2 mg), and the dry weight was recorded. Brain water content was calculated as follows: [(wet weight − dry weight) / wet weight] × 100% [50].

### Distribution of aRMCs

aRMCs were adjusted to a concentration of 1 × 10^6^ cells/ml in phosphate-buffered saline (PBS) and labeled with 5 μmol/l DiR dye (Uelandy, Suzhou, China) at 37 °C for 20 min. After incubation, cells were washed twice with prewarmed (37 °C) serum-free culture medium by centrifugation. Labeling efficiency was confirmed by flow cytometry. Labeled or unlabeled aRMCs (1 × 10^6^ cells suspended in saline) were then administered intravenously via the tail vein immediately after reperfusion.

In vivo and ex vivo fluorescence imaging was performed using an IVIS Spectrum system (PerkinElmer, Waltham, MA, USA) to track the distribution of aRMCs at 24 h after administration. Before in vivo imaging, hair on the head and abdomen was removed to minimize light scattering. Animals were subsequently euthanized, and brains and spleens were harvested for ex vivo imaging. After imaging, spleens were frozen, sectioned, and counterstained with DAPI (Servicebio) for microscopic analysis to verify the presence of DiR-labeled aRMCs.

### Isolation of splenocytes

Rats were deeply anesthetized with isoflurane 24 h after surgery. Spleens were aseptically harvested and weighed. Single-cell suspensions were generated by mechanically disintegrating the spleens. Red blood cells (RBCs) were lysed using RBC lysis buffer (BD Biosciences, San Jose, CA, USA). The resulting cells were filtered through 70 μm nylon cell strainers, washed with PBS, and resuspended in RPMI 1640 medium (Thermo Fisher Scientific, Suzhou, China) supplemented with 2% fetal bovine serum (Thermo Fisher Scientific) for further investigation.

### CCK-8 assay for lymphocyte activity

Splenocytes were prepared as described above. Cells were seeded into flat-bottom 96-well plates at a density of 5 × 10^4^ cells/well in 200 μl of medium and cultured at 37 °C in a humidified incubator with 5% CO₂. All conditions were assayed in triplicate. To stimulate proliferation, lipopolysaccharide (LPS, 5 μg/ml, Sigma-Aldrich, St. Louis, MO, USA) was added to activate B lymphocytes, while concanavalin A (Con A, 5 μg/ml, Sigma-Aldrich) was used to activate T lymphocytes. After 48 h of incubation, cell activity was quantified using a Cell Counting Kit-8 (MCE, Shanghai, China). Briefly, 20 μl of tetrazolium substrate solution was added to each well. After a further 4 h incubation under the same culture conditions, the optical density (OD) at 450 nm was measured using an ELISA plate reader (BioTek, Winooski, VT, USA).

### Flow cytometric analysis

Splenocytes were prepared as described above. For T-cell analysis, cells were stained with PerCP/Cyanine5.5-conjugated anti-rat CD3 (Cat. No. E-AB-F1228J, Elabscience, Wuhan, China), PE-conjugated anti-rat CD4 (Cat. No. E-AB-F1105D, Elabscience), and FITC-conjugated anti-rat CD8a (Cat. No. E-AB-F1098C, Elabscience). For monocyte and neutrophil profiling, cells were labeled with PE-conjugated anti-rat CD43 (Cat. No. 202812, BioLegend, San Diego, CA, USA) and FITC-conjugated anti-rat granulocyte marker HIS48 (Cat. No. 11-0570-82, eBioscience, San Diego, CA, USA). Monocyte subsets and neutrophils were identified based on CD43 and HIS48 expression as follows: CD43^-^HIS48^+^ cells were defined as classical monocytes, CD43^+^HIS48^-^ cells as non-classical monocytes, and CD43^+^HIS48^+^ cells as neutrophils [51].

To assess CCR5 expression on T cells, splenocytes were stained with PerCP/Cyanine5.5-conjugated anti-rat CD3, PE-conjugated anti-rat CD4, APC-conjugated anti-rat CD8a (Cat. No. E-AB-F1098E, Elabscience), and FITC-conjugated anti-CCR5 (Cat. No. ab11466, Abcam, Cambridge, UK).

All staining procedures were performed according to the manufacturer’s instructions. Stained cells were analyzed using a NovoCyte 3080 flow cytometer (Agilent Technologies, Santa Clara, CA, USA), and data were processed using FlowJo software (v10.7.1).

### RNA sequencing and analysis

Spleens were harvested and flash-frozen 24 h after surgery. Total RNA was extracted using TRIzol (Invitrogen) and assessed for concentration and integrity using a NanoDrop 2000 (Thermo Fisher Scientific) and Agilent 2100 Bioanalyzer (Agilent Technologies). mRNA was enriched, purified, and fragmented, followed by first- and second-strand cDNA synthesis with end repair and A-tailing. Adapters were ligated, target fragments were purified using VAHTS DNA Selection Beads, and libraries were PCR-amplified. Library quality was checked using the DNA 1000 Assay Kit (Agilent Technologies), and high-throughput sequencing was performed on the Illumina NovaSeq X Plus. Clean reads were mapped to the reference genome using HISAT2, and gene expression levels were quantified and normalized as FPKM and TPM using RSEM.

Differential expression analysis between groups was performed using DESeq2, with genes showing a false discovery rate (FDR) < 0.05 and an absolute fold change ≥ 2 considered as differentially expressed (DEGs). Expression patterns across groups were analyzed using Short Time-series Expression Miner (STEM), functional enrichment, and pathway analyses were performed using Gene Ontology (GO), Kyoto Encyclopedia of Genes and Genomes (KEGG), and gene set enrichment analysis (GSEA). Immune cell abundance was estimated using the quanTIseq algorithm [52].

### H&E and immunofluorescence staining

Rats were deeply anesthetized and transcardially perfused with PBS followed by 4% paraformaldehyde. The spleens and brains were then harvested, post-fixed in 4% paraformaldehyde for 24 h, and embedded in paraffin. Tissue sections were cut at a thickness of 4 μm. Spleen sections were stained with hematoxylin and eosin. Brain sections were subjected to immunofluorescence staining to assess T-cell infiltration. The following primary antibodies were used: recombinant anti-CD3 antibody (Cat. No. GB15014, Servicebio), CD4 rabbit polyclonal antibody (Cat. No. bs-0766R, Bioss, Woburn, MA, USA), and anti-CD8a rabbit polyclonal antibody (Cat. No. GB115692, Servicebio). To identify T-cell subsets, double immunofluorescence staining for CD3⁺CD8⁺, and CD3⁺CD4⁺ cells was performed. After overnight incubation with primary antibodies, sections were incubated with appropriate fluorescent secondary antibodies (Servicebio) and counterstained with DAPI. Images were analyzed with Servicebio Aipathwell and ImageJ software.

### T-cell isolation

Splenocytes were isolated and resuspended in EasySep™ buffer (STEMCELL, Vancouver, BC, Canada). CD3^+^ T cells were isolated by magnetic negative selection according to the manufacturer’s instructions using the EasySep™ Rat T Cell Isolation Kit (STEMCELL). Isolated T cells were subsequently used in functional assays and gene expression analysis. The purity of T lymphocytes was assessed by flow cytometry.

### RT-qPCR

RNA was isolated from T cells (1 × 10^7^ cells) using TRIzol (Invitrogen), and first-strand cDNA was synthesized using a Reverse Transcription kit (Servicebio). mRNA expression levels of specific genes were quantified using a QuantStudio 3 Real-Time PCR System (Thermo Fisher Scientific) with a SYBR Green qPCR Master Mix (Servicebio). The thermocycler conditions were as follows: initial denaturation at 95 °C for 30 s, followed by 40 cycles of 95 °C for 15 s and 60 °C for 30 s. Gene expression levels were normalized to the endogenous control, GAPDH, and relative fold changes were calculated using the 2^-ΔΔCt method.

### In vitro migration assay

T-cell migration was assessed using 24-well Transwell plates with 5 μm polycarbonate membrane pores (Corning, NY, USA) as previously described [53]. The lower chamber of each well was filled with 800 μl of RPMI 1640 medium supplemented with 15% FBS. Subsequently, 0.5 × 10⁶ T cells suspended in 200 μl of serum-free medium were seeded into the upper chamber. To evaluate the role of CCR5, parallel experiments were performed in which the CCR5 antagonist maraviroc (10 μmol/l; Selleck Chemicals, Houston, TX, USA) was added to the cell suspension in the upper chamber. Plates were incubated for 24 h at 37 °C in a humidified atmosphere containing 5% CO₂.

After incubation, the number of migrated T cells in the lower chamber was quantified using an automated cell counter (Halo Counter, Beijing, China). The migration percentage was calculated as follows: Migration percentage = (number of cells in the lower chamber/number of cells seeded in the upper chamber) × 100%. The migration inhibition percentage was calculated as follows: migration inhibition percentage = [1 − (number of cells in the lower chamber with maraviroc/number of cells in the lower chamber without maraviroc)] × 100%.

### Statistical analysis

Clinical data were analyzed using IBM SPSS Statistics version 23.0. The normality of quantitative variables was assessed using the Shapiro–Wilk test. Normally distributed data are presented as mean ± standard deviation (SD), and intergroup comparisons were performed using the independent samples t-test. Non-normally distributed data are presented as the median and interquartile range [M (P25, P75)], and comparisons were conducted using the Mann–Whitney U test. Categorical variables are presented as frequencies and percentages, and intergroup differences were analyzed using the chi-square test. In the univariate analysis, factors with *p* < 0.1 were subjected to binary logistic regression. *p* < 0.05 was considered statistically significant.

Experimental data were expressed as the mean ± SD. Comparisons among multiple groups were performed using one-way analysis of variance (ANOVA) followed by Tukey’s multiple-comparisons test, which checks the variance in comparison groups before performing the analysis. The variance was similar in the comparison groups. Data analysis was conducted with the GraphPad Prism software, and significant differences were defined with *p* < 0.05.

### Additional information

The original data presented in this study are publicly available and can be found at: https://ngdc.cncb.ac.cn/gsa/browse/CRA040625.

## Supplementary information

Supplementary material
