# Supplementary material for: Antler stem cells effectively alleviate the symptoms of cerebral ischemic injury via immunomodulation of the spleen
Source: Cell Death Discov. 2026 Jun 24;12:359. doi: 10.1038/s41420-026-03218-4 (PMC13554090; doi:10.1038/s41420-026-03218-4)
Supplement: Supplementary file 1 [file 41420_2026_3218_MOESM1_ESM.pdf]

Supplementary Information

Antler stem cells effectively alleviate the symptoms of cerebral ischemic injury via immunomodulation of the spleen

Si Wu[1,2], Yu Gao[1], Shunshun Zhong[3], Qianqian Guo[2], Yan Zhang[1], Huiying Li[1], Jiping Li[2], Huiling Luo[5], Hongyu Zhang[3], Qing Leng[3], Yufang Shi*[4], Zhonghui Liu*[3], Chunyi Li*[2], Guangxian Nan*[1]

[1]Department of Neurology, China-Japan Union Hospital of Jilin University, Changchun, 130033, P.R. China.

[2]Institute of Antler Science and Product Technology, Changchun Sci-Tech University, Changchun, 130600, P.R. China.

[3]Department of Immunology, College of Basic Medical Sciences, Jilin University, Changchun, 130021, P.R. China.

[4]The Fourth Affiliated Hospital of Soochow University, Institutes for Translational Medicine of Soochow University, Suzhou, 215123, P.R. China.

[5]Department of Cardiology, China-Japan Union Hospital of Jilin University, Jilin Provincial Cardiovascular Research Institute, Changchun, 130033, P.R. China.

*Corresponding authors: Yufang Shi, email: yfshi@suda.edu.cn; Zhonghui Liu, email: liuzh@jlu.edu.cn; Chunyi Li, email: lichunyi1959@163.com; Guangxian Nan, email: nangx@jlu.edu.cn.

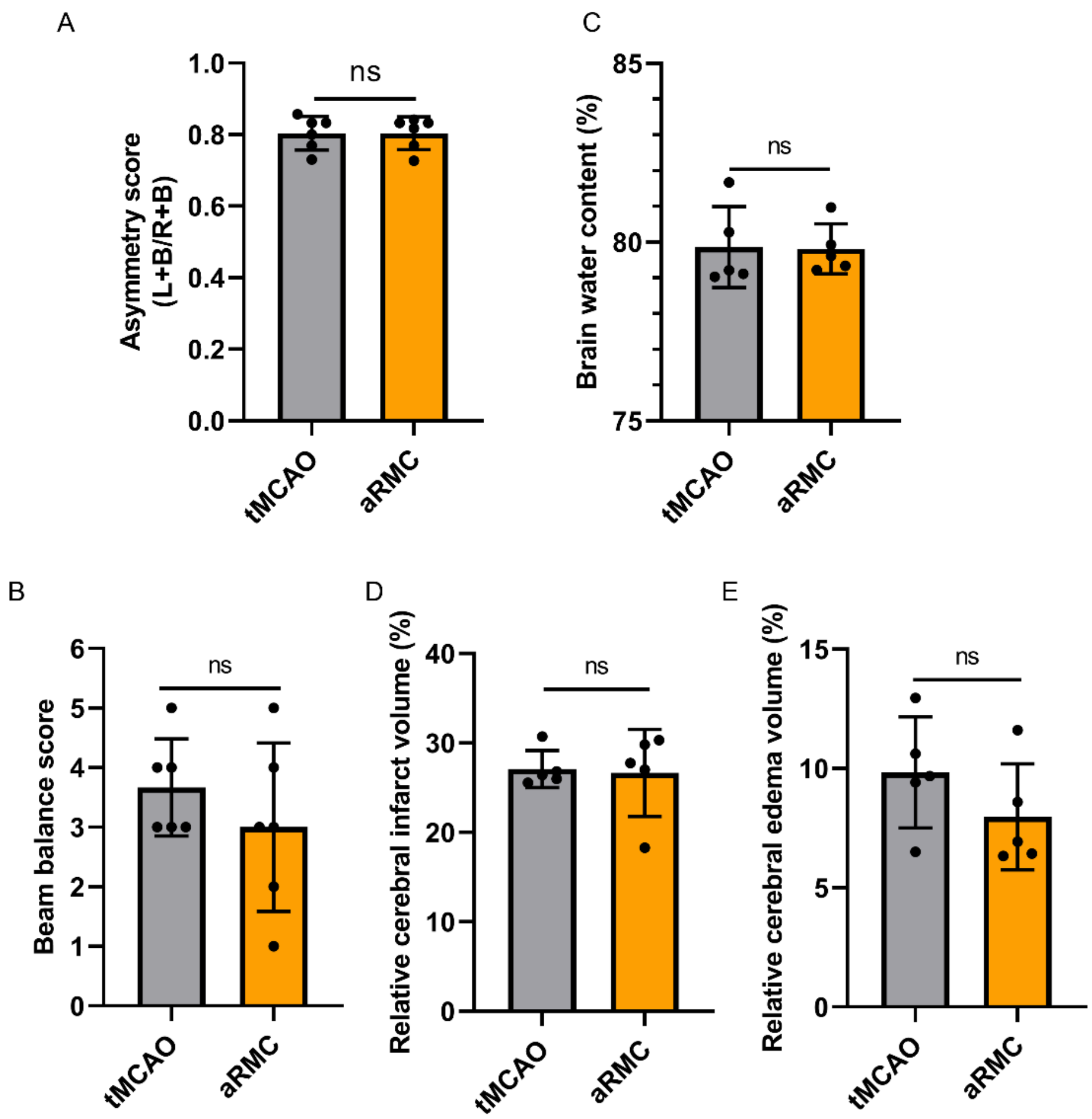

Fig. 1 Assessment of behavioral outcomes, infarct volume, and brain water content in rats with splenectomy. A Functional recovery assessed using the cylinder test (n = 6). B, both left and right forelimbs; L, left forelimb; R, right forelimb. **B** Functional recovery assessed using the beam balance test (n = 6). **C** Brain water content assessment (n = 5). **D**

Representative TTC-stained brain sections. **E**, **F** Relative cerebral infarct volume and cerebral edema volume were calculated based on TTC staining (n = 5). Data expressed as mean ± SD. *$p < 0.05$, **$p < 0.01$, ***$p < 0.001$, ns non-significant.

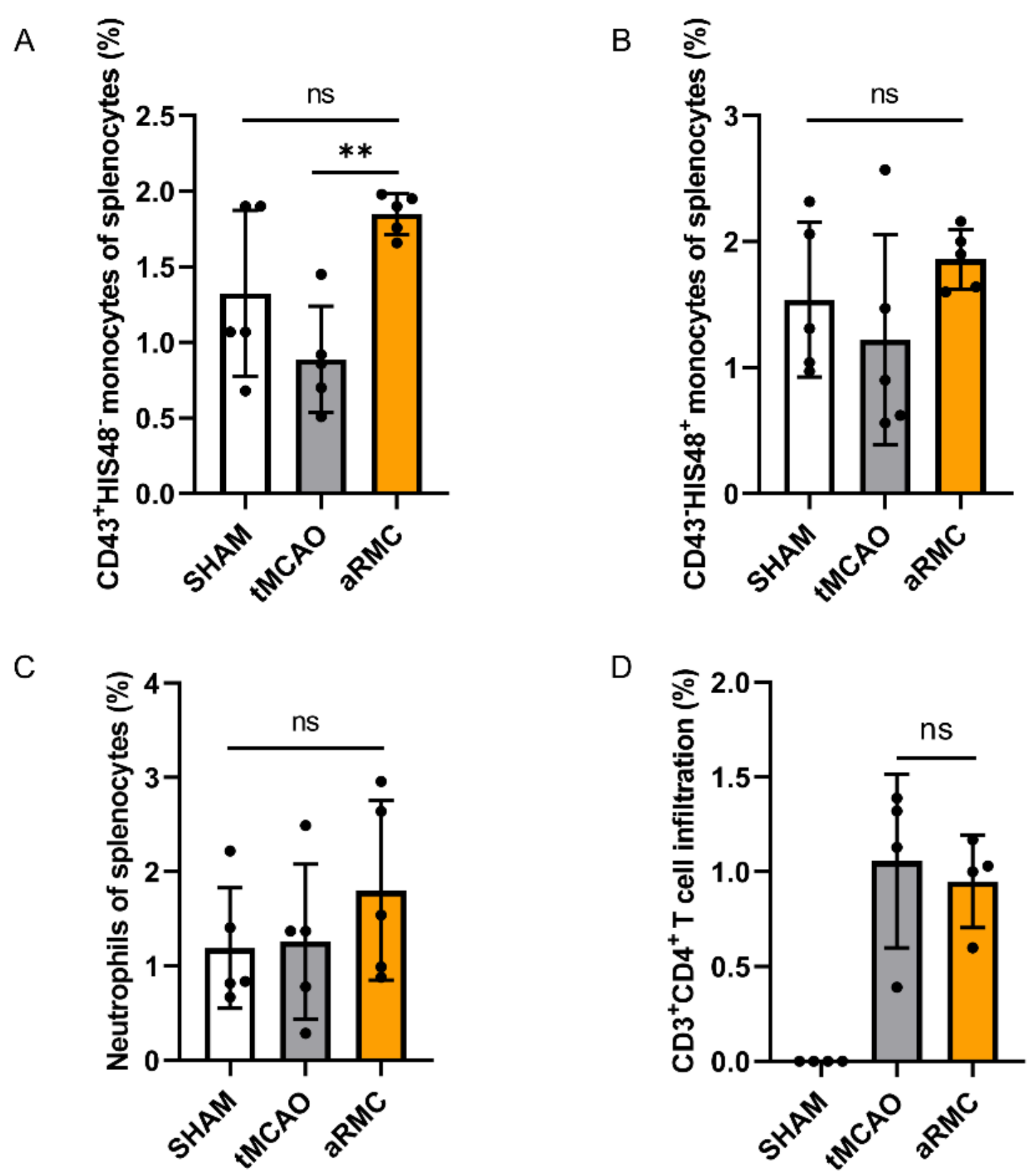

Fig. 2 Splenic immune cell composition and brain T-cell infiltration. A Flow cytometric analysis of $CD43^{+}HIS48^{-}$ monocytes among splenocytes (n = 5). **B** Flow cytometric analysis of $CD43^{-}HIS48^{+}$ monocytes among splenocytes (n = 5). **C** Flow cytometric analysis of $CD43^{+}HIS48^{+}$ neutrophils among splenocytes (n = 5). **D** Quantification of

$CD3^{+}CD4^{+}$ T-cell infiltration in cerebral infarct region ($n = 4$). Data expressed as mean ± SD. $*p < 0.05$, $**p < 0.01$, $***p < 0.001$, ns non-significant.

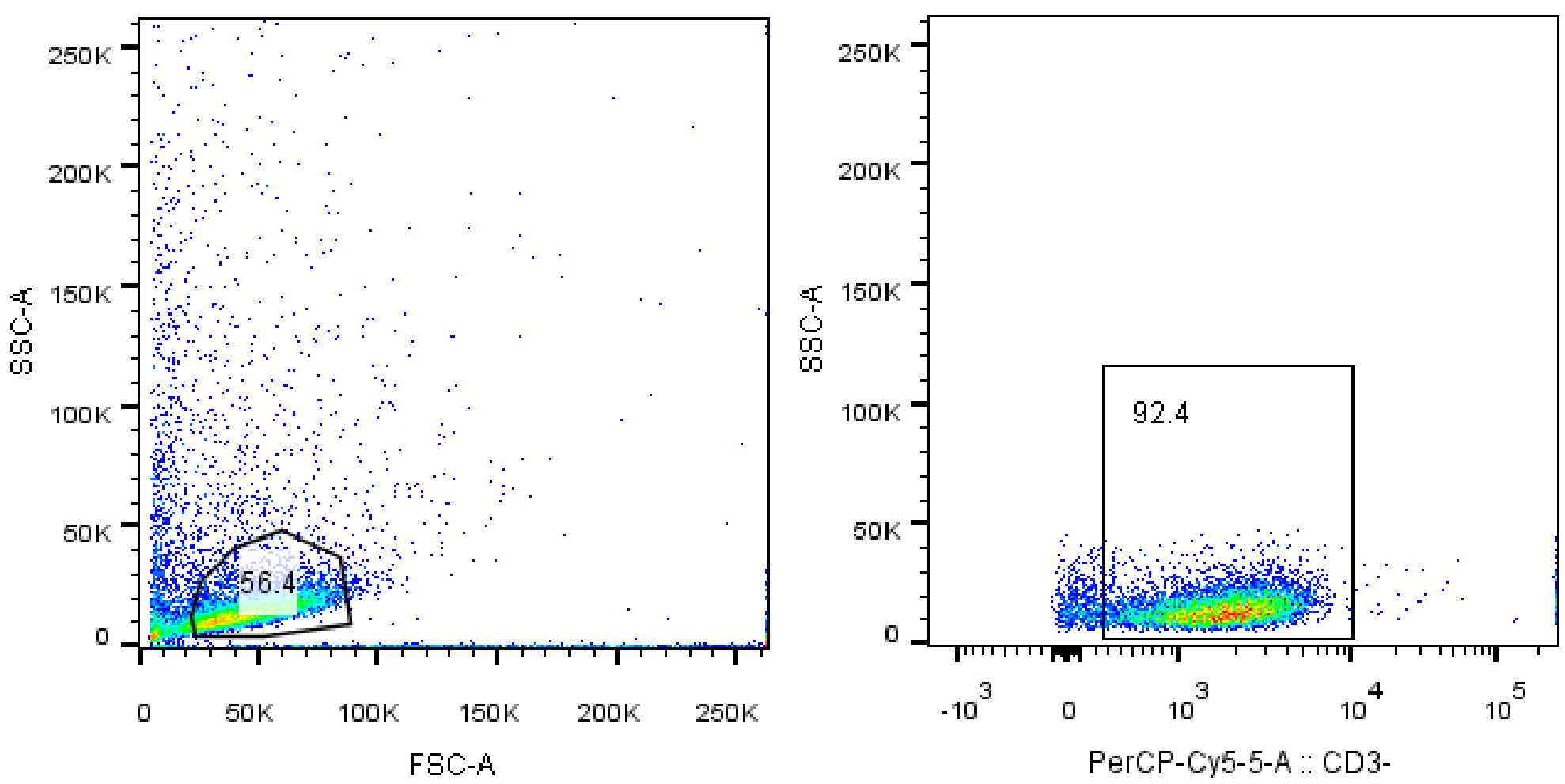

Fig. 3 The purity of T lymphocytes isolated from splenocytes was 92.4%.

Table 1. Main characteristics of patients with and without spleen volume reduction

	Spleen Volume Reduction (n = 25)	No Spleen Volume Reduction (n = 51)	Z/t, X^2	p-value
**Baseline characteristics**				
Age, yrs	65.28±12.99	66.84±10.30	0.569	0.571
Male sex, n (%)	16 (64%)	36 (70.59%)	0.337	0.562
**Clinical characteristics**				
Hypertension, n (%)	15 (60%)	24 (47.06%)	1.125	0.289
Diabetes, n (%)	7 (28%)	14 (27.45%)	0.003	0.96
Atrial fibrillation, n (%)	8 (32%)	8 (15.69%)	2.686	0.101
Cerebral infarction/TIA, n (%)	4 (16%)	16 (31.37%)	2.045	0.153
White blood cell (WBC) count ($\times 10^9$/l)	8.14 (IQR 6.49, 9.91)	9.04 (IQR 7.01, 11.29)	-1.382	0.167
Blood neutrophil count ($\times 10^9$/l)	6.83 (IQR 4.43, 8.45)	6.89 (IQR 5.34, 8.65)	-0.719	0.472
Blood lymphocyte count ($\times 10^9$/l)	0.93 (IQR 0.75, 1.53)	1.35 (IQR 0.96, 1.96)	-2.134	0.033
Blood monocytes count ($\times 10^9$/l)	0.33 (IQR 0.23, 0.40)	0.41 (IQR 0.26, 0.55)	-2.051	0.04
NIHSS-admission	17 (IQR 12.5, 20)	15 (IQR 12, 18)	-0.821	0.412
Edema expansion, n (%)	18 (72%)	16 (31.37%)	11.2	0.001

Table 2. Binary logistic regression analysis of brain edema expansion

Covariate	OR value	95% CI	p value
Age	0.964	0.918, 1.012	0.143
Cerebral infarction/TIA	0.441	0.124, 1.572	0.207
NIHSS-admission	1.112	1.002, 1.233	0.046
Spleen volume reduction	5.862	1.872, 18.358	0.002

Table 3. Primer sequences for genes

Gene	Primer Sequence
GAPDH	FORWARD: ACAGTCCATGCCATCACTGCC
	REVERSE: GCCTGCTTCACCACCTTCTTG
Il1b	FORWARD: ACAGCAGCATCTCGACAAGAGC
	REVERSE: CCACGGGCAAGACATAGGTAGC
Ccr5	FORWARD: CAGGCAATGCAGGTGACAGAGAC
	REVERSE: CCTGTGGACCGGGTATAGACTGAG
Vcam1	FORWARD: ACTGTGACCTGTCAGCGAAG
	REVERSE: TTAGGGACCGTGCAGTTGAC
Nck1	FORWARD: TGGCACAGCAGGAACAAGAGC
	REVERSE: CGAACTCGCCACCAGGACTTAG
Ptprc	FORWARD: GCTGAGCATTGCCCACAAAA
	REVERSE: GGTCTTCAGGAACCCCATGG
Sdc4	FORWARD: GTGTCCATGTCCAGCACTTCCC
	REVERSE: CTACGCCGCCCACAATCAGAG
Tlr4	FORWARD: TGTATCGGTGGTCAGTGTGC
	REVERSE: CAGCTCGTTTCTCACCCAGT
Jag1	FORWARD: TCAACCGGGGAACATGTAGC
	REVERSE: ACTCACACTCAAAGCCCGAG
Ccr3	FORWARD: CCCACACCCACTGCTGTATCAATC
	REVERSE: CCAGTTTCTCGCCAGGAAGGAATG
